# Efficacy of Intra-Arterial Plus Intravesical Chemotherapy for High-Risk Non-Muscle-Invasive Bladder Cancer: A Pooled Analysis

**DOI:** 10.3389/fphar.2021.707271

**Published:** 2021-09-16

**Authors:** Chunliang Cheng, Dongxu Qiu, Jinbo Chen, Xiongbing Zu, Jinhui Liu, Huihuang Li, Jiao Hu, Zhenglin Yi, Tongchen He, Zhi Chen, Yu Cui

**Affiliations:** Department of Urology, Xiangya Hospital, Central South University, Changsha, China

**Keywords:** intra-arterial chemotherapy, intravesical chemotherapy, high-risk non-muscle-invasive bladder cancer, recurrence, progression

## Abstract

**Background:** The treatment for high-risk non-muscle-invasive bladder cancer (NMIBC) remains highly debated for its high recurrence and progression risk. This work aimed to verify the efficacy and toxicity of intra-arterial chemotherapy (IAC) plus intravesical chemotherapy (IVC) in high-risk NMIBC.

**Methods:** A comprehensive online literature search was conducted in three databases to select researches related to IAC + IVC for high-risk NMIBC. All data were analyzed using the Review Manager software version 5.3. And we used the Cochrane Risk of Bias tool to assessed the quality of these enrolled researches.

**Results:** Seven eligible original publications were enrolled in our studies with a total of 1,247 patients. Compared with the intravesical instillation, IAC + IVC therapy showed a better therapeutic effect. The total odds ratio for tumor recurrence rate, tumor progression rate, survival rate, and tumor-specific death rate was calculated as 0.51 (95% CI: 0.36–0.72; *p* < 0.05), 0.51 (95% CI: 0.36–0.72; *p* < 0.05), 1.75 (95% CI: 1.09–2.81; *p* < 0.05), and 0.48 (95% CI: 0.28–0.84; *p* < 0.05), respectively. In patients who received IAC, most of the adverse events (AEs)in the treatment were Grade I and II.

**Conclusion:** IAC + IVC regimen for high-risk NMIBC could effectively reduce recurrence and progression and provide a better prognosis than intravesical instillation. The adverse events of IAC were mild and acceptable.

## Introduction

Bladder cancer (BCa) is the ninth most common cancer worldwide, with approximately 430,000 new cases every year (Antoni et al., 2017). BCa is a heterogeneous disease with different subtypes and molecular classifications, which have distinct treatments, response to drugs and prognosis. Clinically, it can be divided into muscle-invasive bladder cancer (MIBC) and non-muscle-invasive bladder cancer (NMIBC). Patients with NMIBC routinely undergo bladder-sparing surgery with (without) subsequent intravesical installation. Compared with MIBC, NMIBC is generally associated with a better prognosis. However, even with comprehensive management, it has the potential to progress into MIBC, especially high risk NMIBC. Generally speaking, NMIBC can be subdivide into three risk groups (low-risk, intermediate-risk, and high-risk tumors). High-risk NMIBC is defined as any of the following: T1 tumor, G3 (HG) tumor, carcinoma *in situ* (CIS), multiple, recurrent, and large (>3 cm) TaG1G2 /LG tumors (all features must be present) (Babjuk et al., 2019).

However, the treatment protocol for high-risk NMIBC remains highly disputed. Intravesical chemotherapy is widely used in the treatment of NMIBC, such as gemcitabine, mitomycin C, epirubicin, and doxorubicine. However, intravesical chemotherapy for high-risk NMIBC is proven to be inferior to BCG in complete response (OR 0.53, *p* = 0.0002) (Sylvester et al., 2005), and tumor progression (OR 0.73, *p* = 0.001) (Sylvester et al., 2002). Therefore, in recent years transurethral tumor resection with subsequent intravesical BCG instillation has been considered the gold standard of bladder-sparing therapy by EAU guidelines (Babjuk et al., 2013). Notably, over 50% of patients used BCG would gradually develop into BCG failure, which greatly increases the risk of cancer progression (Sylvester et al., 2002; Schrier et al., 2004). Nevertheless, patients who receive BCG carried a high risk of side effects such as cystitis, frequency, and haematuria (Koch, Smelser, and Chang 2020). Because of BCG unresponsiveness and shortage, there is a need to explore effective alternatives as options. In consideration of high-risk progression, radical cystectomy is considered as an option for high-risk NMIBC. However, it would be an overtreatment and deeply reduce patients’ quality of life (QOL) with significant morbidity (Huang et al., 2019a; De Berardinis et al., 2011). In addition, intravesical chemotherapy would be viable option for patients who are refractory to BCG or refuse cystectomy (Dalbagni et al., 2006). Some chemotherapy agents have shown specific promise in BCG-failure patients (eg., gemcitabine, thermochemotherapy, taxane chemotherapy), while some have been shown to be effective only in non-BCG-failure cohorts (e.g., electromotive mitomycin) (Yates et al., 2012).

As a novel drug delivery, intra-arterial chemotherapy was first designed by Kubota in 1989 (Chen et al., 2013). It was performed to insert an angiographic catheter through femoral arteries into vessels adjacent to bladder tumor, and then inject chemotherapeutic agents to increase concentration at tumor site and reduce systemic toxicity (Hoshi et al., 1997; Huang et al., 2019a). IAC has shown its efficacy in reducing disease recurrence and progression for MIBC, which would be a remedial treatment for MIBC patients following TURBT (Liang et al., 2015). In recent years, the IAC + IVC regimen after TURBT in T1-staged Grade 3 (T1G3) high-risk NMIBC patients was reported to reduce the risk of recurrence and progression in several studies (Huang et al., 2019b; Zhang et al., 2016; Chen et al., 2013). However, a retrospective study found that IAC + IVC may fail to decrease progression in the NMIBC patients (Lian et al., 2019). Besides, all present studies were single institution trial with limited patient number that had not strong enough evidence to verify its therapeutic efficacy in high-risk NMIBC. Therefore, in this work, we purposed to provide a more comprehensive report to evaluate the IAC + IVC regimen’s efficacy after TURBT in high-risk NMIBC.

## Materials and Methods

This pooled analysis was conducted according to the items in the Preferred Reporting Items for Systematic Reviews and Meta-analyses (PRISMA) guidelines, and registered with INPLASY (ID: 10.37766/inplasy 2021.1.0031).

### Search Strategy

A comprehensive online literature search was conducted in PubMed, Embase, and the Cochrane Library in December 2020, to identify all the researches related to IAC + IVC for high-risk NMIBC. The search terms included (((((((((bladder cancer) OR (non-muscle invasive)) OR (Superficial)) OR (Early)) OR (Ta)) OR (T1)) OR (Tis)) OR (CIS)) AND ((((((intra-arterial chemotherapy) OR (Intra-arterial infusion)) OR (Intra-arterial therapy)) OR (Intra-arterial injection)) OR (gemcitabine)) OR (cisplatin))) AND (((((((((Intravesical perfusion) OR (Intravesical chemotherapy)) OR (Intravesical therapy)) OR (Intravesical irrigation)) OR (BCG)) OR (Bacille Calmette-Guérin)) OR (epirubicin)) OR (pirarubicin)). All studies on this topic were reviewed, and related references of original studies were identified by manual search.

### Inclusion and Exclusion Criteria

Seven eligible studies were selected in accordance with the PICOS principle. 1) population:patients with pathologically confirmed high-risk NMIBC; 2) intervention: patients treated with IAC + IVC regimen; 3) comparison: treated with intravesical installation alone; 4) outcomes: prognosis indicators including tumor recurrence rate, tumor progression rate, survival rate, tumor-specific death rate, adverse events and time to first tumor recurrence; 5) study design: any studies related to this subject including prospective or retrospective studies or randomized controlled trial. Eligible studies should meet the following criteria:1) patients underwent bladder-preserving surgery 2) pathologically confirmed high-risk NMIBC 3) interventions were IAC + IVC 4) sufficient data that includes tumor recurrence rate, tumor progression rate, survival rate, or cancer-specific survival 5) prospective or retrospective cohort studies or randomized controlled trial 6) published with the English language. Studies involving radical cystectomy, neoadjuvant treatment, immunotherapy, and unsuitable types such as reviews, case reports, editorials, and letters were excluded.

### Data Extraction

Two reviewers (Chunliang Cheng and Dongxu Qiu) were allotted to assess the six eligible publications and extract the data independently. Any conflicts would resort to the judgment by the third reviewer (Jinhui Liu). The detailed data we extracted included the authors’ name, publication year, country, study design, patient number, gender number, age, tumor stage, IAC and IVC regimen, duration of follow-up, event number, and oncologic outcomes.

### Quality Assessment

Two independent reviewers (Huihuang Li and Jiao Hu) used the Cochrane Risk of Bias tool to assessed the quality of the literature. The assessment focused on the potential risk of bias as following: random sequence generation (selection bias), allocation concealment (selection bias), blinding of participants and personnel (performance bias), blinding of outcome assessment (detection bias), incomplete outcome data (attrition bias), selective reporting (reporting bias), and other biases. All studies were manually classified into four evidence quality levels: high, moderate, low, and very low. For any disagreement, the risk of bias was solved by consensus. Besides, publication bias was evaluated through visual inspection of funnel plots, and a sensitivity analysis was conducted using the leave-one-out cross-validation.

### Statistical Analysis

This pooled-analysis was performed to identify the therapeutic efficacy of the IAC + IVC regimen for patients with pathologically diagnosed with high-risk NMIBC by comparing odds ratio, 95% CIs of tumor recurrence rate, tumor progression rate, survival rate, tumor-specific death rate. And AEs extracted or calculated from the enrolled seven studies. I^2^ test was used to assess the eligible studies’ heterogeneity (I^2^ < 25%: no heterogeneity; I^2^ = 25–50%: moderate heterogeneity; I^2^ > 50%: large heterogeneity). A fixed-effect model would be used when the I^2^ < 50% and a random effect model would be applied when I^2^ > 50%. Toxicities associated with IAC were evaluated according to Common Terminology Criteria for Adverse Events, version 4.0 (CTCAE v4.0). All analyses were performed by using Review Manager version 5.3 (The Nordic Cochrane Centre, The Cochrane Collaboration, Copenhagen), all *p*-values were two-sided, and the level of statistical significance was considered at *p* < 0.05.

## Results

### Study Selection and Characteristics

**FIGURE 1:**
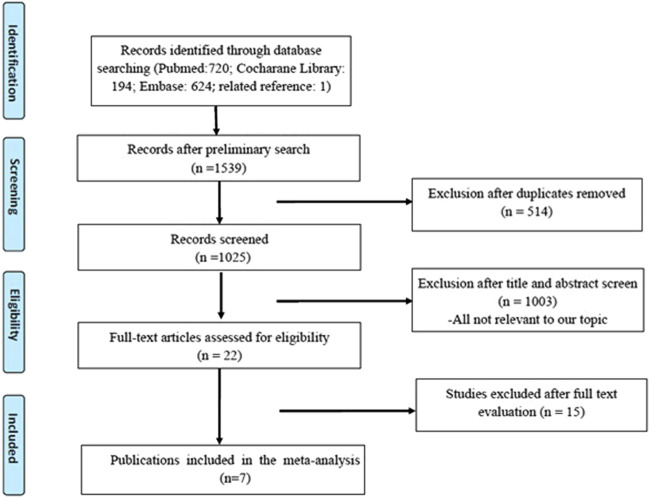
Flowchart for article selection.

A total of 1,539 original publications were retrieved through systematic search, among them 514 were excluded as repeated records. After manual selection for titles, abstracts, and full-text articles, we enrolled seven eligible studies involving 1,247 patients who are pathologically diagnosed with NMIBC after bladder-preserving surgery (Chen et al., 2013; Lian et al., 2019; Huang et al., 2019b; Huang et al., 2019a; Sun et al., 2017; Zhang et al., 2016; J; Huang et al., 2020). The detailed process of the studies’ selection was shown in Figure 1.

Among these researches, three of them was prospective studies, and all the studies was performed in the Chinese hospital. In total, 511 high risk NMIBC patients received IVC and subsequent IAC regimen, while 736 underwent intravesical installation alone. Most of the studies administered similar IAC regimen (cisplatin plus epirubicin or pirubicin), and the interval time between each cycle ranged from 3 to 6 weeks. The detailed characteristics and drug usage of each enrolled study was listed in Table 1. And the final pooled results of all studies were displayed in Table 2.

**TABLE 1:** Characteristics of included studies.

Author	Country	Type	Patient number C/M	Stage	Regime in C	Regime in M	IAC	IVI	Follow-upMonths C/M	Outcome
Chen et al. (2013)	China	prospective single center	29/31	T1G3	IAC: epirubicin + cisplatin IVI: epirubicin	epirubicin	Cisplatin (60 mg/m2) and epirubicin (50 mg/m2); Once every 4–6 weeks;	Epirubicin (50 mg/m2) immediately after TURBT; Weekly for 8 weeks and monthly for 8 months;	22/23	TRR, TPR, SR, TSD, AEs
Huang et al. (2018)	China	prospective single center	69/131	T1G3	IAC: cisplatin + epirubicin IVI: pirarubicin	pirarubicin	Cisplatin (60 mg/m2) and epirubicin (50 mg/m2); Four times with 1-month interval;	Pirarubicin immediately after TURBT; Weekly for 8 weeks and monthly for 10 months;	79/59	TRR, TPR, SR, TSD, AEs
Huang et al. (2018)	China	retrospective single center	53/98	T1G3	IAC: cisplatin (60 mg/m2) + pirarubicin (50 mg/m2) IVI: epirubicin	epirubicin	Cisplatin (60 mg/m2) and pirarubicin (50 mg/m2); Four times with 1-month interval;	Pirarubicin immediately after TURBT; Weekly for 8 weeks and monthly for 10 months;	98/82	TRR, TPR, SR, TSD, AEs
Huang et al. (2020)	China	retrospective single center	43/53	High risk NMIBC	IAC: cisplatin + epirubicin or pirarubicinIVI: Epirubicin or pirarubicin	BCG	cisplatin (60 mg/m2) and epirubicin (50 mg/m2); four courses with an internal of 1 month	IVI in C: once a week until 8 weeks and once a month until 10 months; IVI in M: once a week in the first 6 weeks, once every 2 weeks in the next 6 weeks, once a month until 10 months.	28/25	TRR, TPR, SR, TSD, AEs
Lian et al. (2019)	China	retrospective single center	69/32	Ta-T1 (only extract patients with high risk NMIBC)	IAC: cisplatin + epirubicin IVI: epirubicin	epirubicin	Cisplatin (60 mg/m2) and pirubicin (50 mg/m2); Once every 4–6 weeks; Three cycle;	Epirubicin (50 mg/50 ml) immediately after TURBT; Weekly for 4–8 weeks and monthly for 6–12 months;	24.25/22.30	TRR
Sun et al. (2017).	China	prospective single center	141/142	High risk NMIBC	IAC: cisplatin + epirubicin IVI: epirubicin	epirubicin	Cisplatin (50 mg/m2) and epirubicin (30 mg/m2); Three courses at 4-week intervals;	Cisplatin (50 mg/m2) and epirubicin (30 mg/m2); Three courses at 4-week intervals;	47.3/46.8	TRR, TPR, SR, TSD, AEs
Zhang et al. (2016)	China	retrospective single center	176/281	T1G3	IAC: gemcitabine + oxaliplatin	epirubicin or pirarubicin	gemcitabine (1,200 mg/m2) plus oxaliplatin (100 mg/m2) for every 21 days, at least two cycles	1–2 weeks after TURBT and proceeded once weekly for 8 weeks, then monthly for 12 months	33/33	TRR, TPR, AEs

C, combined therapy group M; monotherapy group TRR, tumor recurrence rate; TPR, tumor progression rate; SR, survival rate; TSD, tumor-specific death rate; AEs, adverse events.

**TABLE 2:** Summary of therapeutic efficacy and survival outcomes.

Outcomes	No of participants (studies)	No. of patients (events)		Effect relative (95% CI)	P	I^2^	Effect model
		IAC + IVC	Monotherapy				
TRR	7 studies	150	309	OR 0.51 (0.40–0.65)	<0.05	41%	Fixed
TPR	6 studies	54	135	OR 0.51 (0.36–0.72)	<0.05	31%	Fixed
SR	5 studies	307	385	OR1.75 (1.09–2.81)	<0.05	0%	Fixed
TSD	5 studies	18	53	OR 0.48 (0.28–0.84)	<0.05	10%	Fixed

TRR, tumor recurrence rate; TPR, tumor progression rate; SR, survival rate; TSD, tumor-specific death rate; AEs, adverse events; OR, odds ratio; CI, confidence interval.

### Tumor Recurrence Rate

**FIGURE 2:**
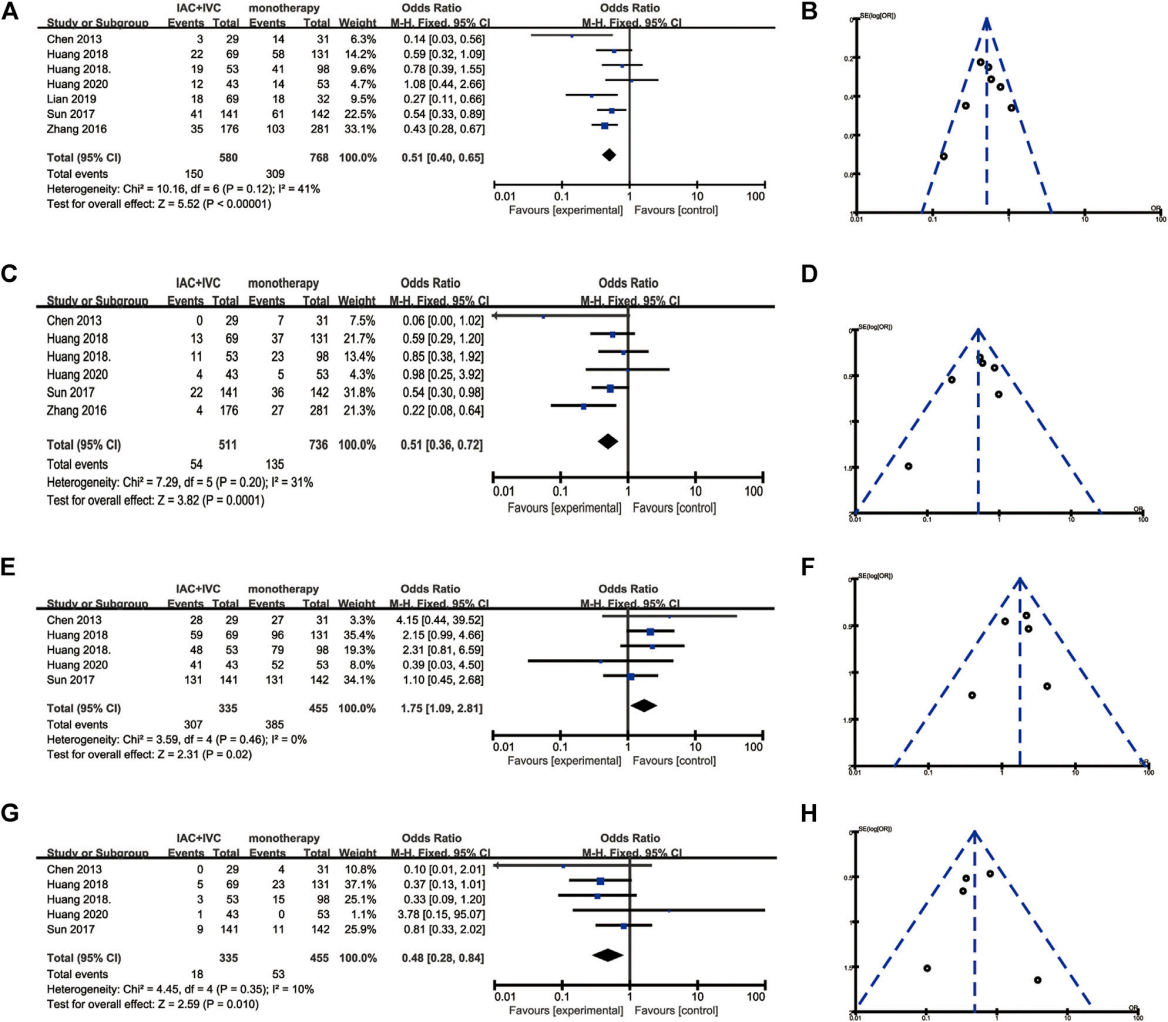
Forest plots and funnel plots of IAC + IVC vs. intravesical installation. **(A)** Forest plot of tumor recurrence rate, **(B)** Funnel plot of tumor recurrence, **(C)** Forest plot of tumor progression rate, **(D)** Funnel plot of tumor recurrence, **(E)** Forest plot of survival rate, **(F)** Funnel plot of survival rate, **(G)** Forest plot of tumor-specific death rate, **(H)** Funnel plot of tumor-specific death rate.

Totally, all seven studies reported the tumor recurrence rate (Chen et al., 2013; Lian et al., 2019; Huang et al., 2019b; Huang et al., 2019a; Sun et al., 2017; Zhang et al., 2016; J. Huang et al., 2020; Chen et al., 2013). 25.9% (150/580) patients in the IAC + IVC group and 40.2% (309/768) in the monotherapy group had tumor recurrence. The pooled OR for recurrence rate was calculated as 0.51 (95% CI:0.40–0.65; *p* < 0.05) which showed IAC + IVC had a superiority in inhibiting recurrence. Because the heterogeneity (I^2^ < 50%) was moderate for tumor recurrence rate, a fixed-effect model was used (Figure 2A).

### Tumor Progression Rate

Tumor progression rate results were extracted in six enrolled studies (Huang et al., 2020; Huang et al. 2019b; Huang et al. 2019a; Sun et al., 2017; Zhang et al., 2016; J.; Chen et al., 2013), 10.6% (54/511) patients in IAC + IVC group had progression compared with 18.3% (135/736) in the monotherapy groups. The pooled OR for this result was 0.51 (95% CI: 0.36–0.72; *p* < 0.05). It indicated that IAC + IVC could also significantly inhibit tumor progression in high risk NMIBC. There was no significant heterogeneity among studies based on Cochran Q statistics (Figure 2C).

### Survival Rate

Five studies reported survival data (Chen et al., 2013; Huang et al. 2019b; Huang et al. 2019b; Huang et al. 2019a; Huang et al., 2020). Among all the studies, 91.6% (307/335) high risk NMIBC patients in the IAC + IVC group survived at the end of follow-up compared with 84.6% (385/455) in monotherapy group. The pooled OR for survival rate was calculated as 1.75 (95% CI: 1.09–2.81; *p* < 0.05), which implied that the IAC + IVC regimen provided a better survival. Based on the Cochran Q statistics (*p* < 0.05), there was no heterogeneity among studies when I^2^ = 0% (Figure 2E).

### Tumor-specific Death Rate

Five of the enrolled studies reported tumor-specific death rate data (Huang et al., 2020; Huang et al. 2019b; Huang et al. 2019a; Sun et al., 2017;; Chen et al., 2013). Among all the studies, 5.4% (18/335) high risk NMIBC patients in the IAC + IVC group died from bladder cancer before the end of follow-up compared with 11.6% (53/455) in monotherapy group. The pooled OR for this result was calculated as 0.48 (95% CI: 0.28–0.84; *p* < 0.05), which represented a significantly lower tumor-specific death in the IAC + IVC group. There was no heterogeneity among studies as the I^2^ = 10% with the application of the Cochran Q statistics (*p* < 0.05) (Figure 2G).

### Quality Assessment, Sensitivity, and Publication Bias

**FIGURE 3:**
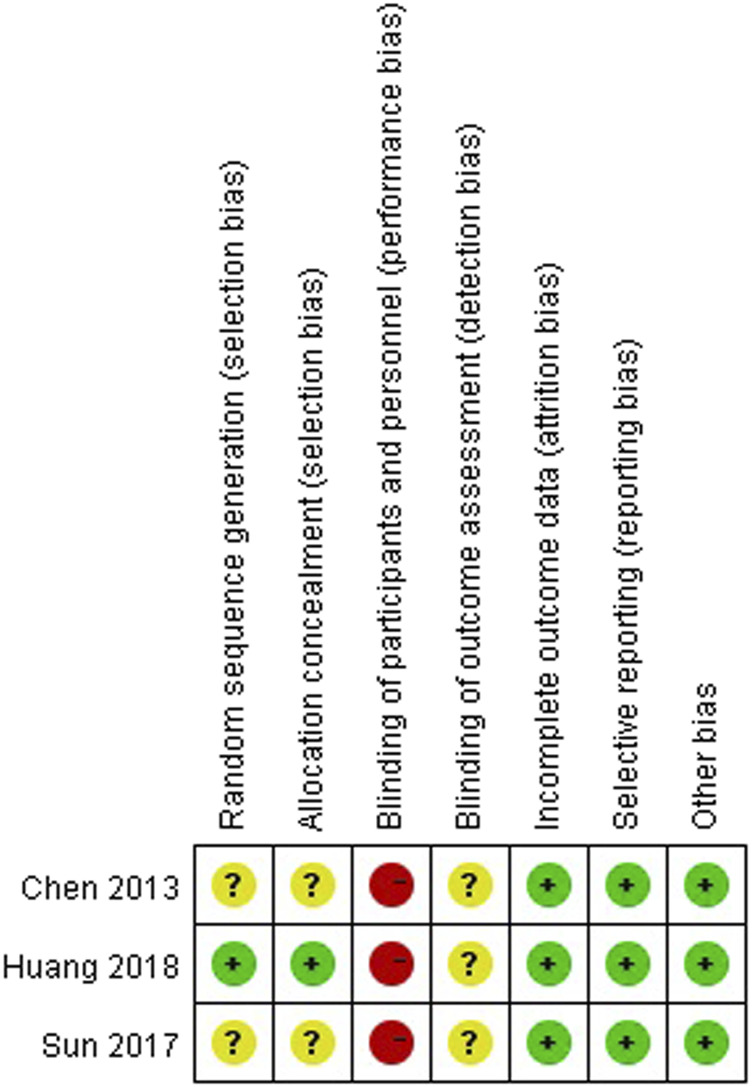
Risk of bias graph depicting each risk of bias item as percentages across prospective studies.

**FIGURE 4:**
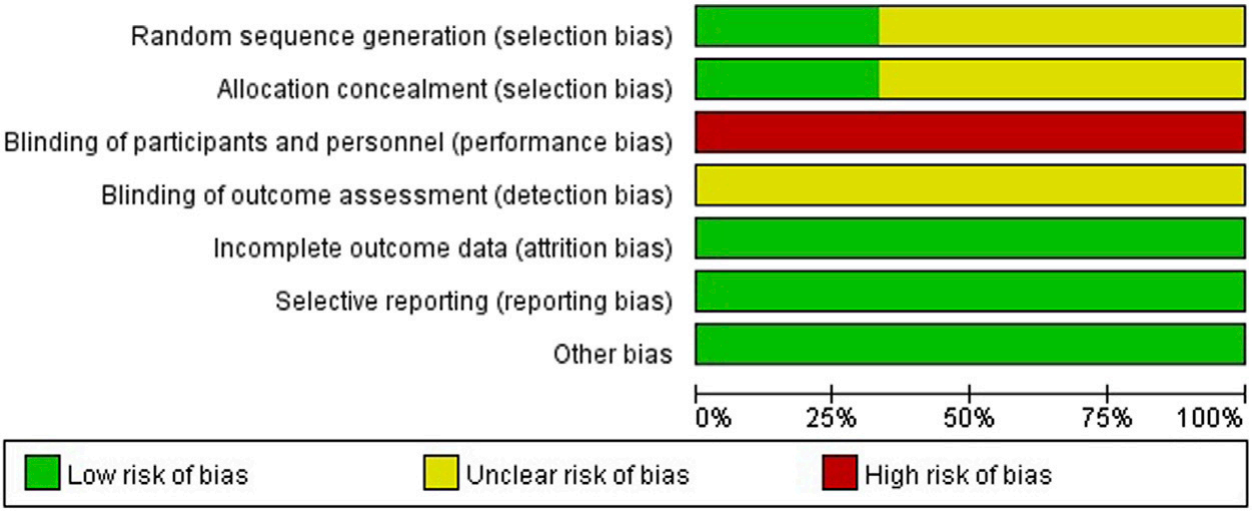
Risk of bias summary.

To verify how individual studies influenced the pooled results, a sensitivity analysis was performed by eliminating one study at a time. No serious publication bias was found according to the funnel plots (Figures 2B,D,F,H). Among all the enrolled publications, three of them were prospective trials (42.9%) (Huang et al., 2019a; Sun et al., 2017; Chen et al., 2013). Therefore, we utilized Cochrane Collaboration’s risk of the bias assessment tool to assess the risk of bias in these three studies. All the three studies reported to use the randomization methods, but two of them did n not mention details (Sun et al., 2017; Chen et al., 2013). Only one studies described allocation concealment (Huang et al., 2019a), and none of them reported blind method in the researches. The assessment of risk of the bias was shown in Figures 3, 4.

### Subgroup Analysis

**FIGURE 5:**
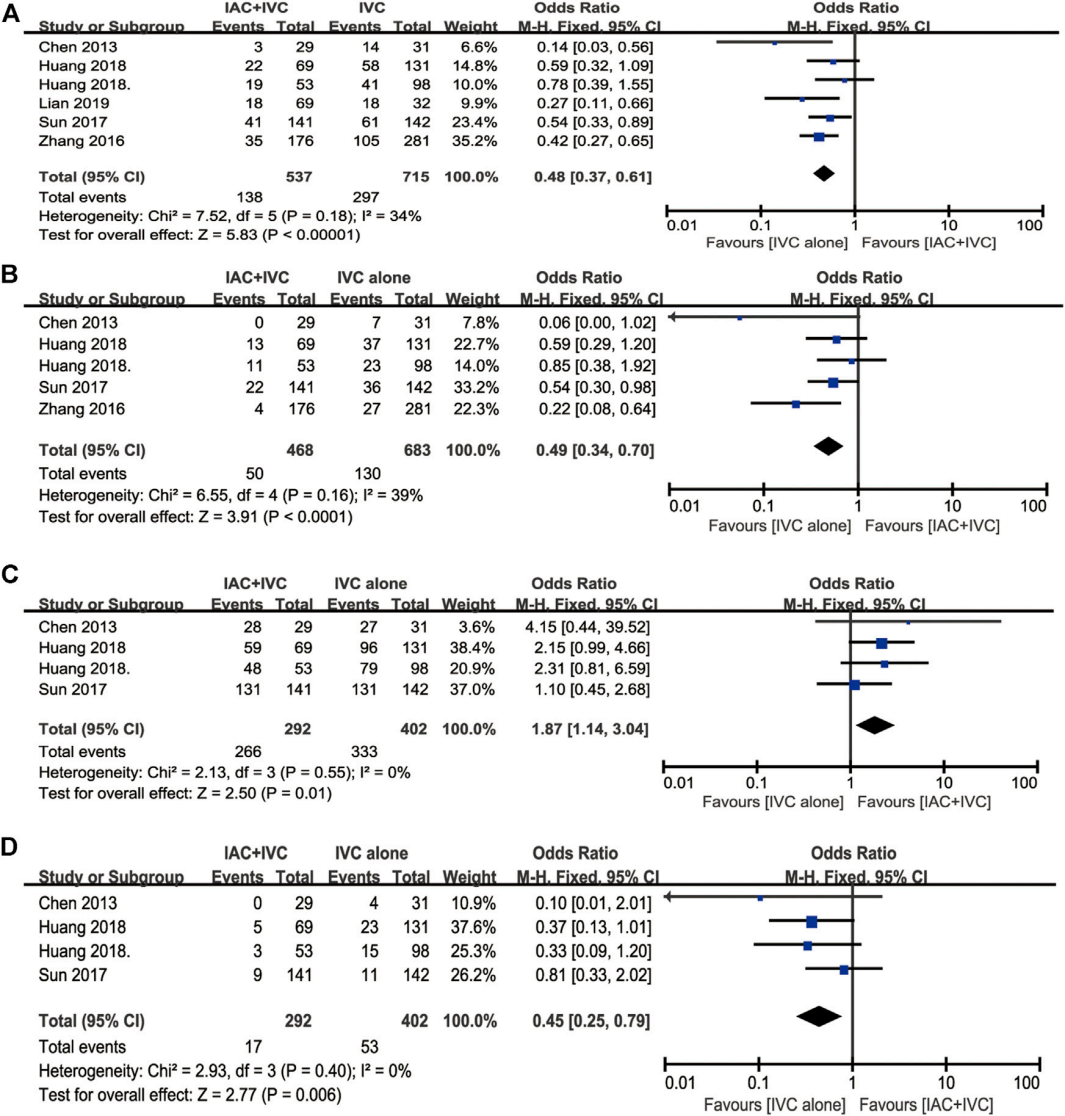
Subgroup analysis for IAC + IVC vs. IVC. **(A)** tumor recurrence rate, **(B)** tumor progression rate, **(C)** survival rate, **(D)** tumor-specific death rate.

We noticed, in this analysis, six studies used IVC alone in the monotherapy group (Chen et al., 2013; Huang et al. 2019a; Huang et al. 2019b; Zhang et al., 2016; Sun et al., 2017; Huang et al., 2020). Therefore, we made this subgroup analysis to compare the efficacy of IAC + IVC with IVC alone. The pooled OR for tumor recurrence rate, tumor progression rate, survival rate, and tumor-specific death rate for these six studies was calculated as 0.48 (95% CI: 0.37–0.61; *p* < 0.05), 0.49 (95% CI:0.34–0.70; *p* < 0.05), 1.87 (95% CI:1.14–3.04; *p* < 0.05), 0.45 (95% CI:0.25–0.79; *p* < 0.05), respectively, (Figure 5).

### Toxicity of IAC

**FIGURE 6:**
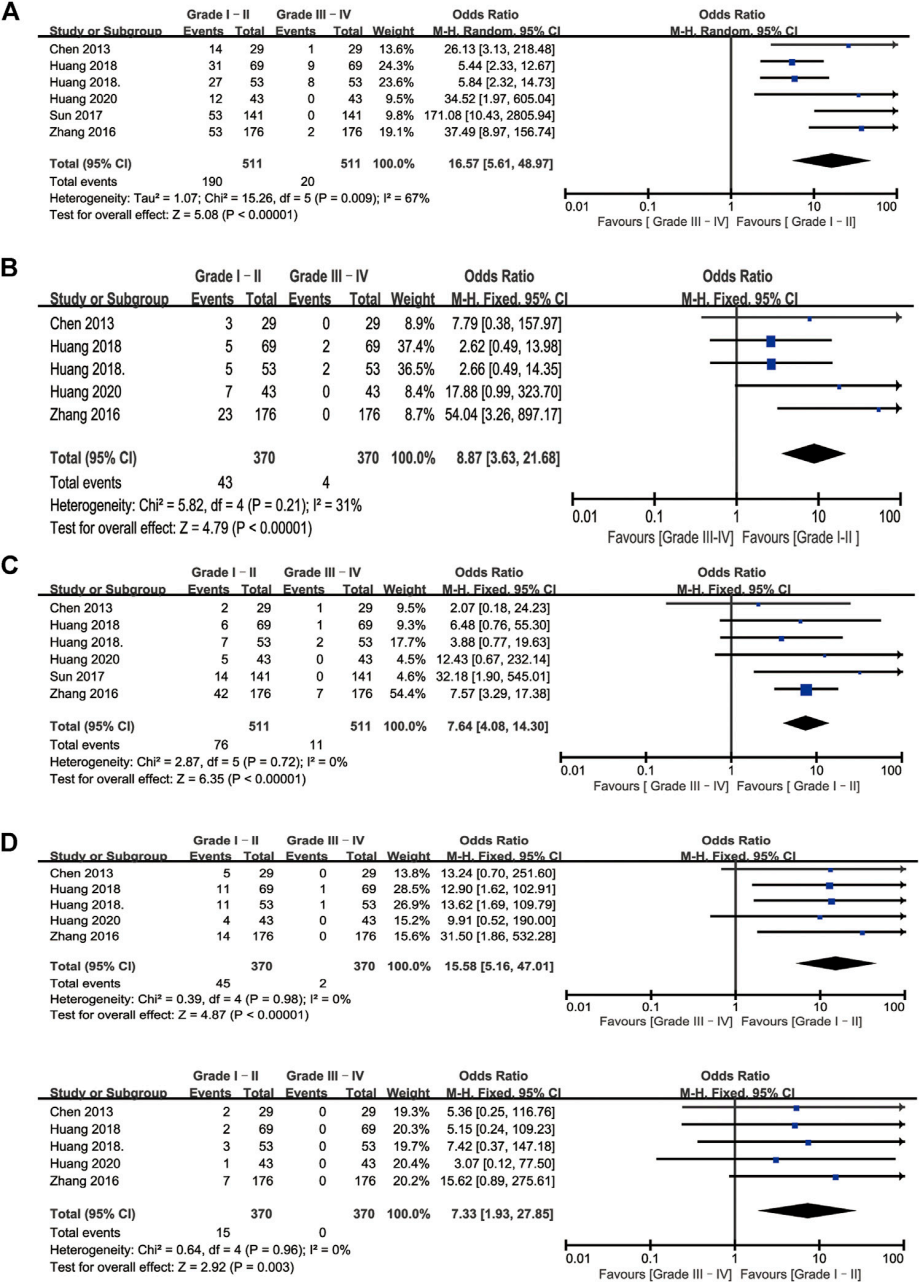
Forest plots of complications associated with IAC. **(A)** Nausea/vomiting **(B)** Hypoleukemia **(C)** Neutropenia **(D)** Increased alanine aminotransferase **(D)** Increased creatinine.

A total of six studies reported toxicities data after IAC treatment (Huang et al., 2020; Huang et al. 2019b; Huang et al. 2019a; Sun et al., 2017; Zhang et al., 2016; J.; Chen et al., 2013). Among these studies, the most frequently reported adverse effects (AEs) were nausea/vomiting, hypoleukemia, neutropenia, increased alanine aminotransferase, and increased creatinine. Other AEs in the IAC group could be seen in (Zhang et al., 2016; Sun et al., 2017), such as fever, constipation/diarrhea, anemia, thrombocytopenia, and pain. No local reaction such as puncture haematoma, allergy was observed or mentioned, and no patients died from treatment-related complications. The detailed AEs data was displayed in Table 3. Besides, we also made a forest plot to compare the severity of each complication. According to CTCAE v4.0, AEs related to IAC was divided into two groups. AEs on Grade I–II was regarded as mild and easily reversible complications. As shown in Figure 6, compared with Grade III–IV, Grade I-II was significantly common in nausea/vomiting, hypoleukemia, neutropenia, increased alanine aminotransferase, and increased creatinine.

**TABLE 3:** Adverse reactions of intra-arterial chemotherapy.

Side effect	Study	Grade I–II	Grade III–IV	Incidence %
Nausea/vomiting	Chen et al. (2013)	14	1	51.7
	Huang et al. (2018)	31	9	57.9
	Huang et al. (2018)	27	8	66.0
	Huang et al. (2020)	12	0	27.9
	Sun et al. (2017)	53	0	37.6
	Zhang et al. (2016)	53	2	31.2
Hypoleukemia	Chen et al. (2013)	3	0	10.3
	Huang et al. (2018)	5	2	11.3
	Huang et al. (2018)	5	2	13.2
	Huang et al. (2020)	7	0	16.3
	Zhang et al.(2016)	23	0	13.1
Neutropenia	Chen et al. (2013)	2	1	10.3
	Huang et al. (2018)	6	1	11.3
	Huang et al. (2018)	7	2	16.9
	Huang et al. (2020)	5	0	11.6
	Sun et al. (2017)	14	0	9.9
	Zhang et al. (2016)	42	7	27.8
Increased alanine aminotransferase	Chen et al. (2013)	5	0	17.2
	Huang et al. (2018)	11	1	17.4
	Huang et al. (2018)	11	1	22.6
	Huang et al. (2020)	4	0	9.3
	Zhang et al. (2016)	14	0	7.95
Increased creatinine	Chen et al. (2013)	2	0	6.90
	Huang et al. (2018)	2	0	2.3
	Huang et al. (2018)	3	0	4.5
	Huang et al. (2020)	1	0	2.3
	Zhang et al. (2016)	7	0	3.98
Fever	Huang et al. (2020)	4	0	9.3
Constipation/diarrhea	Zhang et al. (2016)	40	3	24.4
	Sun et al. (2017)	26	1	22.7
Anemia	Sun et al. (2017)	20	1	14.9
Thrombocytopenia	Sun et al. (2017)	9	0	6.4
Pain	Sun et al. (2017)	8	0	5.7

According to CTCAE v4.0 (Common Terminology Criteria for Adverse Events, version 4.0).

## Discussion

The treatment for high-risk NMIBC remains highly debated by urologists for its high risk of recurrence and progression. Currently, the recommended options to treat high-risk NMIBC include immediate RC and bladder-sparing therapy followed by comprehensive treatment (Babjuk et al., 2013). Although adjuvant intravesical therapy with full-dose intravesical BCG for 1–3 years following bladder-sparing therapy is currently regarded as the gold standard for high-risk NMIBC, the recurrence and progression rate were 38.6 and 9.8% in the NMIBC patients who received BCG intravesical therapy (Huang et al., 2020). Besides, not all patients are able to receive the 1–3 years of treatment recommended in current guidelines because BCG intravesical therapy is associated with more side effects such as cystitis, frequency, haematuria, and fever, and these side effects would not decrease even though with the usage of one-third dose (Brausi et al., 2014). Thus, there are various emerging bladder sparing therapies as options for patients who do not respond to BCG in recent years (Packiam et al., 2019).

At first, intra-arterial chemotherapy, as novel drug delivery, was primarily used as neoadjuvant chemotherapy for muscle-invasive bladder cancer (MIBC) (Maatman et al., 1986; Kakizaki et al., 1987). And then, IAC has shown favorable results when combined with irradiation for MIBC (Sumiyoshi et al., 1994; Miyanaga et al., 2000; Hata et al., 2006). Recently, IAC was proven to be more effective than IVC in preventing high-risk superficial bladder cancer from recurrence and progression with a 44.4% recurrence-free survival rate and 75.4% progression-free survival rate in several studies (Liu et al., 2018; M.K.; Chen et al., 2009). Furthermore, IAC + IVC was reported to reduce the risk of recurrence and progression with lower toxicities (Huang et al., 2019a; Chen et al., 2013); however, the oncological benefit still needs to be verified.

To our knowledge, it is the first time that there is an evaluation of the efficacy of the IAC + IVC regimen in high-risk NMIBC through a pooled-analysis. In this pooled analysis involving 1,247 high-risk NMIBC patients, 25.9% (150/580) patients in the IAC + IVC group and 40.2% (309/768) in the IVC group had tumor recurrence. Moreover, IAC + IVC’s overall superiority versus IVC in reducing tumor recurrence was calculated as 0.51 (95% CI:0.40–0.65; *p* < 0.05). As a reference, Bohle et al. in his meta-analysis of 11 eligible clinical trials, found a statistically significant lower recurrence rate for BCG installation (38.6%) compared with the mitomycin IVC (46.4%) (Böhle et al., 2003). The recurrence rate for IVC in our analysis 40.2% (309/768) was similar to 46.4% in Bohle et al., whereas the recurrence rate for IAC + IVC 25.9% (150/580) was obviously lower than BCG installation (38.6%). It indicated that IAC + IVC was significantly superior to IVC and may even better than BCG in reducing tumor recurrence. On the other hand, in our pooled analysis, 10.6% (54/511) patients in IAC + IVC group had progression compared with 18.3% (135/736) in the IVC groups. The reduction in odds ratio of progression was 49% (OR: 0.51 95% CI: 0.36–0.72; *p* < 0.05). The progression rate in our study for IAC + IVC (10.6%) was similar with BCG (9.8%) in a meta-analysis conducted by Sylvester et al. (Sylvester et al., 2002), which indicates that IAC + IVC have a lower progression compared with IVC and would be as effective as BCG in reducing tumor progression.

Besides, the survival rate and tumor-specific death rate was calculated as 1.75 (95% CI: 1.09–2.81; *p* < 0.05), 0.48 (95% CI: 0.28–0.84; *p* < 0.05), respectively. The results indicated IAC + IVC have a better prognosis compared with IVC. The heterogeneity of our pooled results for tumor recurrence rate and tumor progression rate was I^2^ = 41% and I^2^ = 31%, respectively. It presented that our pooled results had a moderate heterogeneity. The heterogeneity was potentially caused by some possible reasons, such as 1) patients in the included studies were received different IAC + IVC chemotherapy agents. 2) The number of high-risk NMIBC patients in the present studies were limited, and most of them were retrospective studies.

Toxicity was an important consideration when clinicians formulated a therapeutic regimen. From all the pooled studies, the AEs of IAC were gastrointestinal distress, myelotoxicity, and hepatic and renal dysfunction, most of which were slight adverse reactions (Grade I and II). No common puncture-related arterial complications were observed in all the studies. However, descriptions for technical difficulties were not mentioned in all of enrolled studies. In addition, the GRADE system was used to validate the accuracy of our findings.

However, there still existed some limitations in this study. Firstly, most studies included were retrospective, and a multicenter clinical trial with a larger sample size will still be needed. Secondly, in this pooled analysis, patients in seven studies received diverse chemotherapy agents with different follow-up periods and pathologically diagnosed NMIBC types, which were associated with different risks of recurrence and progression. Thirdly, all the enrolled studies were performed in only three hospitals in China, and lacked data from western countries. Finally, this pooled analysis primarily compared IAC + IVC with IVC, and studies directly contrasting the efficacy with BCG should be further explored.

## Conclusion

The IAC + IVC regimen for high risk-NMIBC could effectively reduce recurrence and progression and provide a better prognosis compared with the IVC monotherapy group. However, considering the potential limitations of this study, more well-performed, multicenter, and larger-scale RCTs are required to verify the value of IAC.

## Data Availability

The original contributions presented in the study are included in the article/supplementary material, further inquiries can be directed to the corresponding authors.

## References

[B1] Antoni S. Ferlay J. Soerjomataram I. Znaor A. Jemal A. Bray F. (2017). Bladder Cancer Incidence and Mortality: A Global Overview and Recent Trends. Eur. Urol. 71 (1), 96–108. 10.1016/j.eururo.2016.06.010 27370177

[B2] Babjuk M. Burger M. Compérat E. M. Gontero P. Mostafid A. H. Palou J. (2019). European Association of Urology Guidelines on Non-muscle-invasive Bladder Cancer (TaT1 and Carcinoma *In Situ*) - 2019 Update. Eur. Urol. 76 (5), 639–657. 10.1016/j.eururo.2019.08.016 31443960

[B3] Babjuk M. Burger M. Zigeuner R. Shariat S. F. van Rhijn B. W. G. Compérat E. (2013). EAU Guidelines on Non-muscle-invasive Urothelial Carcinoma of the Bladder: Update 2013. Eur. Urol. 64 (4), 639–653. 10.1016/j.eururo.2013.06.003 23827737

[B4] Böhle A. Jocham D. Bock P. R. (2003). Intravesical bacillus Calmette-Guerin versus Mitomycin C for Superficial Bladder Cancer: a Formal Meta-Analysis of Comparative Studies on Recurrence and Toxicity. J. Urol. 169 (1), 90–95. 10.1016/s0022-5347(05)64043-8 12478111

[B5] Brausi M. Oddens J. Sylvester R. Bono A. van de Beek C. van Andel G. (2014). Side Effects of Bacillus Calmette-Guérin (BCG) in the Treatment of Intermediate- and High-Risk Ta, T1 Papillary Carcinoma of the Bladder: Results of the EORTC Genito-Urinary Cancers Group Randomised Phase 3 Study Comparing One-Third Dose with Full Dose and 1 Year with 3 Years of Maintenance BCG. Eur. Urol. 65 (1), 69–76. 10.1016/j.eururo.2013.07.021 23910233

[B6] Chen J. Yao Z. Qiu S. Chen L. Wang Y. Yang J. (2013). Comparing Intra-arterial Chemotherapy Combined with Intravesical Chemotherapy versus Intravesical Chemotherapy Alone: a Randomised Prospective Pilot Study for T1G3 Bladder Transitional Cell Carcinoma after Bladder-Preserving Surgery. Cardiovasc. Intervent Radiol. 36 (6), 1521–1526. 10.1007/s00270-013-0594-2 23511989

[B7] Chen M. K. Qin Z. K. Zhou F. J. Han H. Liu Z. W. Li Y. H. (2009). Intra-arterial Chemotherapy Is Reliable in Preventing High-Risk Superficial Bladder Cancer from Recurrence and Progression. J. Chemother. 21 (6), 681–686. 10.1179/joc.2009.21.6.681 20071293

[B8] Dalbagni G. Russo P. Bochner B. Ben-Porat L. Sheinfeld J. Sogani P. (2006). Phase II Trial of Intravesical Gemcitabine in Bacille Calmette-Guérin-Refractory Transitional Cell Carcinoma of the Bladder. Jco 24 (18), 2729–2734. 10.1200/jco.2005.05.2720 16782913

[B9] Hata M. Miyanaga N. Tokuuye K. Saida Y. Ohara K. Sugahara S. (2006). Proton Beam Therapy for Invasive Bladder Cancer: a Prospective Study of Bladder-Preserving Therapy with Combined Radiotherapy and Intra-arterial Chemotherapy. Int. J. Radiat. Oncology*Biology*Physics 64 (5), 1371–1379. 10.1016/j.ijrobp.2005.10.023 16580495

[B10] Hoshi S. Mao H. Takahashi T. Suzuki K. Nose M. Orikasa S. (1997). Internal Iliac Arterial Infusion Chemotherapy for Rabbit Invasive Bladder Cancer. Int. J. Urol. 4 (5), 493–499. 10.1111/j.1442-2042.1997.tb00292.x 9354953

[B11] Huang B. Huang G. Li W. Chen L. Mao X. Chen J. (2020). Intra-arterial Chemotherapy Combined with Intravesical Chemotherapy Compared with Intravesical BCG Immunotherapy Retrospectively in High-Risk Non-muscle-invasive Bladder Cancer after Transurethral Resection of the Bladder Tumor. J. Cancer Res. Clin. Oncol. 147, 1781–1788. 10.1007/s00432-020-03453-x 33222014 PMC11801937

[B12] Huang B. Wang H. Lin H. Yao Z. Zheng J. Fan W. (2019a). Evaluation of the Effects of Intra-arterial Chemotherapy Combined with Intravesical Chemotherapy against Intravesical Chemotherapy Alone after Transurethral Resection of Bladder Tumor in T1-Staged Grade 3 Bladder Cancer. J. Cancer Res. Clin. Oncol. 145 (2), 487–494. 10.1007/s00432-018-2811-5 30539282 PMC11810394

[B13] Huang B. Zheng J. Yao Z. Fan W. Qiu S. Chen L. (2019b). Efficacy of Intra-arterial Chemotherapy Combined with Intravesical Chemotherapy in T1G3 Bladder Cancer when Compared with Intravesical Chemotherapy Alone after Bladder-Sparing Surgery: a Retrospective Study. World J. Urol. 37 (5), 823–829. 10.1007/s00345-018-2437-x 30191393

[B14] Kakizaki H. Suzuki H. Kubota Y. Numasawa K. Suzuki K. (1987). Preoperative One-Shot Intra-arterial Infusion Chemotherapy for Bladder Cancer. Cancer Chemother. Pharmacol. 20 (Suppl. l), S15–S19. 10.1007/bf00262478 3117396

[B15] Koch G. E. Smelser W. W. Chang S. S. (2021). Side Effects of Intravesical BCG and Chemotherapy for Bladder Cancer: What They Are and How to Manage Them. Urology 149, 11–20. 10.1016/j.urology.2020.10.039 33181123

[B16] Lian F. Chen W. Liu Y. Shen L. Fan W. Cui W. (2019). Intra-arterial Chemotherapy Combined with Intravesical Chemotherapy Is Effective in Preventing Recurrence in Non-muscle Invasive Bladder Cancer. J. Cancer Res. Clin. Oncol. 145 (6), 1625–1633. 10.1007/s00432-019-02900-8 30900154 PMC11810414

[B17] Liang S. Zou Q. Han B. Jing Y. Cui D. An X. (2015). Intra-arterial Chemotherapy for Muscle-Invasive Bladder Cancer Following Transurethral Resection. Urol. Int. 94 (4), 406–411. 10.1159/000369301 25678415

[B18] Liu Z. Ye Y. Li X. Guo S. Jiang L. Dong P. (2018). The Effects of Intra-arterial Chemotherapy on Bladder Preservation in Patients with T1 Stage Bladder Cancer. World J. Urol. 36 (8), 1191–1200. 10.1007/s00345-018-2199-5 29459997

[B19] Maatman T. J. Montie J. E. Bukowski R. M. Risius B. Geisinger M. (1986). Intra-arterial Chemotherapy as an Adjuvant to Surgery in Transitional Cell Carcinoma of the Bladder. J. Urol. 135 (2), 256–260. 10.1016/s0022-5347(17)45602-3 3944856

[B20] Miyanaga N. Akaza H. Okumura T. Sekido N. Kawai K. Shimazui T. (2000). A Bladder Preservation Regimen Using Intra-arterial Chemotherapy and Radiotherapy for Invasive Bladder Cancer: a Prospective Study. Int. J. Urol. 7 (2), 41–48. 10.1046/j.1442-2042.2000.00137.x 10710246

[B21] Packiam V. T. Werntz R. P. Steinberg G. D. (2019). Current Clinical Trials in Non-muscle-invasive Bladder Cancer: Heightened Need in an Era of Chronic BCG Shortage. Curr. Urol. Rep. 20 (12), 84. 10.1007/s11934-019-0952-y 31781942

[B22] Schrier B. P. Hollander M. P. van Rhijn B. W. G. Kiemeney L. A. L. M. Alfred Witjes J. (2004). Prognosis of Muscle-Invasive Bladder Cancer: Difference between Primary and Progressive Tumours and Implications for Therapy. Eur. Urol. 45 (3), 292–296. 10.1016/j.eururo.2003.10.006 15036673

[B23] Sumiyoshi Y. Yokota K. Akiyama M. Inoue Y. Yoneda F. Tsujimura H. (1994). Neoadjuvant Intra-arterial Doxorubicin Chemotherapy in Combination with Low Dose Radiotherapy for the Treatment of Locally Advanced Transitional Cell Carcinoma of the Bladder. J. Urol. 152 (2 Pt 1), 362–366. 10.1016/s0022-5347(17)32740-4 8015072

[B24] Sun F. Zhao R. Zhu Y. Cui D. Wang X. Han B. (2017). A Prospective Comparison of Intra-arterial Chemotherapy Combined with Intravesical Chemotherapy and Intravesical Chemotherapy Alone after Transurethral Resection with a Thulium Laser in High-Risk Non-muscle Invasive Bladder Cancer. Cancer Chemother. Pharmacol. 79 (6), 1099–1107. 10.1007/s00280-017-3305-x 28421294

[B25] Sylvester R. J. van der Meijden A. P. M. Lamm D. L. (2002). Intravesical bacillus Calmette-Guerin Reduces the Risk of Progression in Patients with Superficial Bladder Cancer: a Meta-Analysis of the Published Results of Randomized Clinical Trials. J. Urol. 168 (5), 1964–1970. 10.1097/01.ju.0000034450.80198.1c10.1016/s0022-5347(05)64273-5 12394686

[B26] Sylvester R. J. van der Meijden A. P. M. Witjes J. A. Kurth K. (2005). Bacillus Calmette-Guerin versus Chemotherapy for the Intravesical Treatment of Patients with Carcinoma *In Situ* of the Bladder: a Meta-Analysis of the Published Results of Randomized Clinical Trials. J. Urol. 174 (1), 86–91. 10.1097/01.ju.0000162059.64886.1c 15947584

[B27] Yates D. R. Brausi M. A. Catto J. W. F. Dalbagni G. Rouprêt M. Shariat S. F. (2012). Treatment Options Available for Bacillus Calmette-Guérin Failure in Non-muscle-invasive Bladder Cancer. Eur. Urol. 62 (6), 1088–1096. 10.1016/j.eururo.2012.08.055 22959049

[B28] Zhang Y. Xie L. Chen T. Xie W. Wu Z. Xu H. (2016). Intravenous Chemotherapy Combined with Intravesical Chemotherapy to Treat T1G3 Bladder Urothelial Carcinoma after Transurethral Resection of Bladder Tumor: Results of a Retrospective Study. Onco Targets Ther. 9, 605–611. 10.2147/ott.S99866 26869805 PMC4734785

